# Long-Term Effects of Dietary Supplementation with Olive Oil and Hydrogenated Vegetable Oil on the Rumen Microbiome of Dairy Cows

**DOI:** 10.3390/microorganisms9061121

**Published:** 2021-05-22

**Authors:** Nathaly Cancino-Padilla, Natalia Catalán, Karen Siu-Ting, Christopher J. Creevey, Sharon A. Huws, Jaime Romero, Einar Vargas-Bello-Pérez

**Affiliations:** 1 Departamento de Ciencias Animales, Facultad de Agronomía e Ingeniería Forestal, Pontificia Universidad Católica de Chile, Avda. Vicuña Mackenna 4860, Santiago 6904411, Chile; nathaly.cancino@gmail.com; 2 Laboratorio de Biotecnología de Alimentos, Unidad de Alimentos, Instituto de Nutrición y Tecnología de los Alimentos (INTA), Universidad de Chile, El Líbano 5524, Macul, Santiago 7830490, Chile; nataliabcatalant@gmail.com; 3 Institute for Global Food Security, School of Biological Sciences, Queen’s University of Belfast, 19 Chlorine Gardens, Belfast BT9 5DL, UK; agalychnica@gmail.com (K.S.-T.); chris.creevey@qub.ac.uk (C.J.C.); s.huws@qub.ac.uk (S.A.H.); 4 Department of Veterinary and Animal Sciences, Faculty of Health and Medical Sciences, University of Copenhagen, Grønnegårdsvej 3, DK-1870 Copenhagen, Denmark

**Keywords:** rumen, bacteria, 16S rDNA, microbiome, olive oil, palm oil, hydrogenated vegetal oil

## Abstract

Dietary lipids increase energy density in dairy cow diets and in some cases can increase beneficial fatty acids (FA) in milk and dairy products. However, the degree of FA saturation may affect the rumen microbiome. The objective of this study was to determine the long-term effects of feeding saturated (hydrogenated vegetable oil; HVO) or unsaturated (olive oil; OO) fatty acid (FA) sources on the rumen microbiome of dairy cows. For 63 days, 15 mid-lactating cows were fed with either a basal diet (no fat supplement), or the basal diet supplemented with 3% dry matter (DM), either HVO or OO. Rumen contents were collected on days 21, 42 and 63 for 16S rRNA gene sequencing using the Illumina MiSeq platform. The results reveal dominance of the phyla Firmicutes (71.5%) and Bacteroidetes (26.2%), and their respective prevalent genera *Succiniclasticum* (19.4%) and *Prevotella* (16.6%). *Succiniclasticum* increased with both treatments at all time points. *Prevotella* was reduced on day 42 in both diets. Bacterial diversity alpha or beta were not affected by diets. Predicted bacterial functions by CowPI showed changes in energy and protein metabolism. Overall, 3% DM of lipid supplementation over 63 days can be used in dairy cow diets without major impacts on global bacterial community structure.

## 1. Introduction

The rumen microbiome refers to the diversity and function of the community of microorganisms that inhabits the rumen [1]. The rumen microbiome is one of the most diverse ecosystems in nature because it harbors a complex microbial community, composed of anaerobic bacteria, protozoa, fungi, methanogenic archaea and phages [2,3]. These microorganisms play an important role in animal productivity, due to their involvement in the degradation of plant carbohydrates and their subsequent conversion into short-chain fatty acids (volatile fatty acids; VFA), which provide energy for essential metabolic processes [4]. The rumen microbiome also plays a major role in fatty acid metabolism of dietary fats [5]. Typically, dietary forages are high in human health-beneficial polyunsaturated fatty acids (PUFA); nonetheless, the double bonds are removed quickly and efficiently by the rumen bacteria post-ingestion, a process known as biohydrogenation. The rumen bacteria have evolved these mechanisms of removing the double bonds in fatty acids (FA) as they are toxic to them; therefore, their removal ensures their survival, but conversely this leads to ruminant products that are high in human health-detrimental saturated fatty acids (SFA) [5,6]. Nonetheless, the biohydrogenation of dietary FA is often incomplete and intermediate metabolites can reach the duodenum, be absorbed and incorporated into ruminant products, such as milk and meat [7]. Some of these intermediates, such as *cis*-9, *trans*-11 conjugated linoleic acid (CLA), are also beneficial for human health [8,9].

Dairy cow diets can be supplemented with dietary lipids to provide energy for the host and if PUFA-rich they can beneficially increase the human health-beneficial PUFA content of cow’s milk [10,11], cheese [12] and ice cream [13]. For example, supplementation with hydrogenated vegetable oil increases C14:0, C16:0 and C18:0 [13]. It has also been shown that olive oil (OO) by-products can decrease rumen *Anaerovibrio* densities, potentially contributing to a reduction in lipolysis and lowering the availability of PUFA for rumen biohydrogenation in sheep fed on olive oil pomace for 28 days [14]. However, OO can have deleterious effects on the 16S rRNA gene copy numbers of total bacteria when supplemented at 6% DM, as reported in an *in vivo* study, suggesting that this level may impair productivity [15]. 

Longitudinal experiments have been performed for studying temporal variations in microbial communities in beef cattle [16] and during early life in goats [17]. However, there is a lack of knowledge on the long-term effects of supplementing oils on the rumen microbiome of dairy cows. Filling this gap in the current knowledge is important as for years the use of industrial oil by-products has been part of the dairy cow diet, but the impact on rumen microbiota when these feedstuffs are used in the long term remains largely unknown. Therefore, the objective of this study was to determine the effects of dietary fats during a relatively long-term supplementation of either hydrogenated vegetable oil (HVO; as a saturated FA source) or olive oil (OO; as an unsaturated FA source) for 63 days. The hypothesis of this study was that changes in the rumen microbiota would depend on the degree of FA saturation and number of double bonds of dietary fats; therefore, effects should be more noticeable with the use of OO. To ensure contrasting effects, the OO used in this study was composed of 74 g/100 g of C18:1 *cis*-9 whereas HVO was composed mostly of 58 g/100 g of C16:0 and 40 g/100 g of C18:0.

## 2. Materials and Methods

### 2.1. Animal Conditions and Experimental Design

Fifteen multiparous Holstein cows averaging 189 ± 28 days in milk were assigned to three treatment groups based on body condition score (BCS; scored on a five-point scale where 1 = emaciated to 5 = overly fat; [18]) in order to achieve homogeneous experimental groups. At the beginning of the study, the average BCS for the 3 groups were 2.8 ± 0.3, 3.0 ± 0.0 and 2.8 ± 0.3. The study was conducted for 63 days, divided into three periods of 21 days each. All cows received an isocaloric basal restricted diet (NEL = 1.6 Mcal/kg DM) containing 65% forage (corn silage, fresh alfalfa and alfalfa hay) and 35% concentrate (malt distillers, corn grain, wheat bran, soybean grain and rapeseed meal) to satisfy the nutritional requirements of a 650 kg dairy cow in mid-lactation consuming 26.5 kg DM daily [19]. Treatments included a control basal diet with no added lipid (*n* = 5 cows), and a basal diet containing either HVO (*n* = 5 cows; manufactured from palm oil; 3% DM) or OO (*n* = 5 cows; unrefined olive oil residues; 3% DM). Oils were administered separately and mixed manually into the daily ration for each cow. Oils contained the following FA profile: olive oil had 14/100 g of C16:0 and 74/100 g of C18:1 *cis*-9, whereas HVO had 58/100 g of C16:0 and 40/100 g of C18:0. In terms of dietary treatments, OO was composed mainly of C18:0 (26.4/100 g), C18:1 *cis*-9 (32.8/100 g) and C18:2 *cis*-9, *cis*-12 (19/100 g), whereas HVO contained mainly C16:0 (39.2/100 g), C18:0 (30.8/100 g) and C18:2 *cis*-9, *cis*-12 (20/100 g). More details on diets, oils and animals are reported in a companion paper [12]. 

### 2.2. Rumen Samples Analysis

Individual rumen samples were taken on days 21, 42 and 63 using a transesophageal scoop (FLORA; [20]) after morning milking and before feeding. Approximately 15 mL of the liquid fraction containing particulate matter (particles up to 10 mm) was removed from the rumen following Geishauser et al.’s [20] protocol. All technical details on the rumen scoop mechanism, and maintenance while sampling and between samplings have been reported previously [20]. 

Rumen fluid pH was determined immediately after sampling. Samples for ammonia nitrogen (NH_3_-N) determination were centrifuged at 1400× *g* at 4 °C for 20 min and the supernatant was diluted 1:10 with distilled water. Four milliliters of reagent A (50 mg of sodium nitroprusside, 8.25 g of sodium tungstate and 11 mL of 90% liquefied phenol per liter) and reagent B (25 g of disodium phosphate, 5 g of reagent grade sodium hydroxide and 50 mL of 5.25% sodium hypochlorite per liter) were added to 100 μL of rumen fluid. Then, tubes were incubated at room temperature for 1 h and absorbance was subsequently read at 625 nm. Volatile fatty acids (VFA) were determined by gas chromatograph (GC-2010, Shimadzu Scientific Instruments, Columbia, MD, USA) equipped with a 30-m wall-coated open-tubular fused-silica capillary column (Stabilwax-DA; 30 m × 0.32 mm i.d., 0.25 μm film thickness; Restek, Bellefonte, PA, USA). Only samples for VFA determination were preserved with 100 µL 25% metaphosphoric acid. Samples for rumen microbiome were aliquoted in sterile Eppendorf tubes and subsequently stored at −80°C for further analysis.

### 2.3. Rumen Metataxonomic Analysis

Frozen rumen fluid samples were thawed on ice and then homogenized with vortex and 250 mg was weighed in 1.5 mL Eppendorf tubes. Then, 150 μL of PBS (phosphate-buffered saline) was added to each sample in order to perform cell lysis with lysozyme incubation at 37 °C for 60 min, as a pre-treatment for DNA extraction. Consequently, DNA was extracted using the UltraClean Fecal DNA Isolation Kit (MO BIO Laboratories, Carlsbad, CA, USA) according to the manufacturer’s protocol, which involved physical and chemical disruption of cell membranes. Ruminal samples from four animals/treatment were used for sequencing.

The extracted DNA underwent 16S rRNA gene amplification using the bacterial-specific primers 515F 5′-GTGCCAGCMGCCGCGGTAA-3′ and 806R 5′-GGACTACHVGGGTWTCTAAT-3′ [21], to amplify the V3–V4 regions of the 16S rRNA gene. Variable region 4 was selected because sequencing and taxonomic assignment using this region is associated with a low error rate and minimum loss of taxonomic resolution [22]. These primers have been shown to be ideal to amplify the V3–V4 regions with high coverage, and the amplicons (read length) are suitable for the Illumina sequencing platform. Polymerase chain reaction was performed using the following conditions: an initial denaturing cycle of 5 min at 94 °C, followed by 35 cycles of 30 s at 94 °C, annealing at 56 °C for 30 s and an elongation at 68 °C for 45 s. After 16S rDNA V4 region amplification, PCR products were purified through QIAquick PCR Purification kit (Qiagen, Valencia, CA). Subsequently, the purified products were quantified fluorometrically using the High Sensitivity (HS) kit on the Qubit Fluorometer 3.0 (Invitrogen Co., Carlsbad, CA, USA). 

DNA sequencing was performed by CD Genomics (New York, NY, USA) using the Illumina MiSeq 2 × 300 platform. 16S rRNA gene amplicon sequences were quality checked with FASTQC and analyzed using DADA2 and Phyloseq R package version 3.5.1. The quality threshold used for quality filtering of reads was over 28, for forward and reverse reads. Sequences were trimmed to 270 (forward) and 220 bp (reverse). The paired-end Illumina reads were assembled into Amplicon Sequences Variants (ASV) using the DADA2 pipeline. Taxonomy assignation was performed using the Silva training dataset version 132, and sequences corresponding to Eukaryota, Crenarchaeota and Euryarchaeota at the phylum level were removed from the analysis. 

### 2.4. Statistical Analysis

A model including diet, time and diet × time as fixed effects and cow within treatment as a random effect was used to determine differences in animal performance and ruminal fermentation parameters. Analysis of variance (ANOVA) and post-hoc Tukey analysis were per formed using the GenStat (12th edition) statistical package (VSN International Ltd., Oxford, UK). A probability of *p* < 0.05 was considered to indicate a significant difference.

Variations in bacterial relative abundance were determined by two-way ANOVA and Dunnett’s test, comparing the control with lipid-supplemented treatments. Alpha and beta diversity were estimated from the complete bacterial amplicon sequence variant (ASV) table. Alpha (within-sample diversity) and beta diversity (between-sample diversity) measures for samples, grouped by dietary treatments and experimental periods, were analyzed using the phyloseq package in R [23]. Microbial diversity was determined using the Shannon Index (combines richness or the total number of taxa and evenness, the relative abundance of each taxa), dominance was presented using the Simpson index and richness of samples were calculated based on the Chao1 index and observed species. Beta-diversity was calculated using the UniFrac metric and principal coordinates analyses (PCoAs) using both weighted (quantitative) and unweighted (qualitative) Unifrac distances, in order to highlight clusters of similar groups of samples depending on the diet supplementation. In addition, PERMANOVA and PCA were used to elucidate the differences in microbial communities between the three different treatments.

The taxonomic composition of the rumen microbiota was used to predict bacterial function using the CowPI Galaxy Workflow [24], which is a rumen microbiome-focused version of Phylogenetic Investigation of Communities by Reconstruction of Unobserved States (PICRUSt). Raw pathways obtained from CowPi data were categorized according to the KEGG database. Data was blocked by time point and subjected to multiple group ANOVA and Dunnett’s multiple comparisons test using the software GraphPad Prism version 6.00 for Mac OS.

### 2.5. Ethics Statement

The Animal Care Committee of the Pontificia Universidad Católica de Chile approved all the experimental procedures (project ID 150730013), in accordance with their animal care, animal welfare and procedures guidelines, performed at the Estación Experimental Pirque of the Fundación AgroUC (33°38′28″ S, 70°34′27″ W). Animals were housed in individual stalls (2.4 × 6 m) and with ad libitum access to water. 

## 3. Results

### 3.1. Ruminal Fermentation Parameters

Rumen pH, NH_3_-N, total VFA and proportions of individual VFA were similar among treatments (Table 1). From day 21 to 63, total VFA (from 98.5 to 60.8 mmol/L), acetate (66.1 to 64.5 mol/100 mol) and butyrate (from 9.8 to 8.9 mol/100 mol) were decreased, while propionate (from 18.6 to 20.3 mol/100 mol) and iso butyrate (from 1.46 to 2.46 mol/100 mol) were increased. Details on animal performance have been reported previously [12]. Briefly, OO increased milk yield, and reduced milk fat yield, milk fat content and milk somatic cell counts. Compared with control and HVO-supplemented diets, OO decreased C12:0 and increased C18:1 *cis*-9 and C18:3 *cis*-9, *cis*-12, *cis*-15 in milk.

### 3.2. Rumen Metataxonomy

Sequencing the V4 region of the bacterial 16S rRNA gene produced 4,606,204 reads (joined R1–R2 paired-end reads). After data filtering, quality control and chimera removal, a total of 2,104,912 V4 16S rRNA sequence reads from the 48 samples remained, and a mean of 43,852 reads for each sample (minimum, 12,845; maximum, 79,204). Four samples for each treatment and period (48 samples) were used for sequencing. Sequences were trimmed to 270 (forward) and 220 bp (reverse). The amplicon sequence variant (ASVs) identified 43,515 sequences in the rumen of cows fed the control treatment, 44,460 in the rumen of cows supplemented with HVO and 43,583 with OO supplementation (Supp. Mat). A total of 8167 ASV were obtained after analysis with Phyloseq from the bacterial 16S rRNA gene sequencing, which were grouped taxonomically from the phylum to genus level (phylum, class, order, family, genus). 

**Figure 1:**
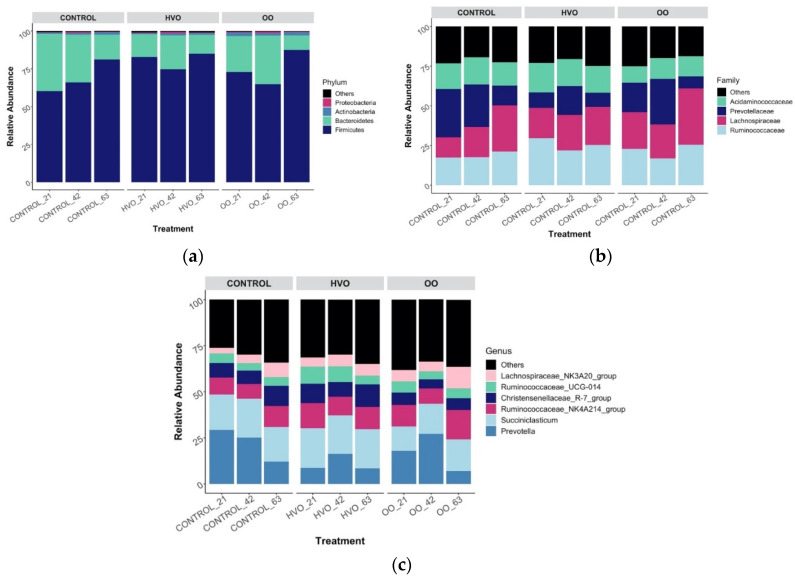
Relative 16S RNA gene abundance at (**a**) phylum-, (**b**) family- and (**c**) genus-level, grouped by dietary treatment, where ‘Others’ correspond to the less abundant (relative abundance ≤ 0.5%).

**Figure 2:**
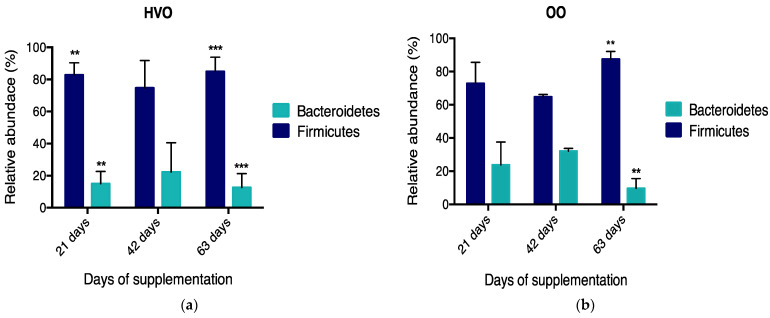
Effect of lipid supplementation and experimental time on relative abundance of the two dominant phyla in rumen microbiota, (**a**) HVO and (**b**) OO. HVO, supplemented with 3% DM hydrogenated vegetable oil; OO, supplemented with 3% DM olive oil. *p*-values were obtained with Dunnett’s multiple comparisons test. ** *p* ≤ 0.01; *** *p* ≤ 0.001.

**Figure 3:**
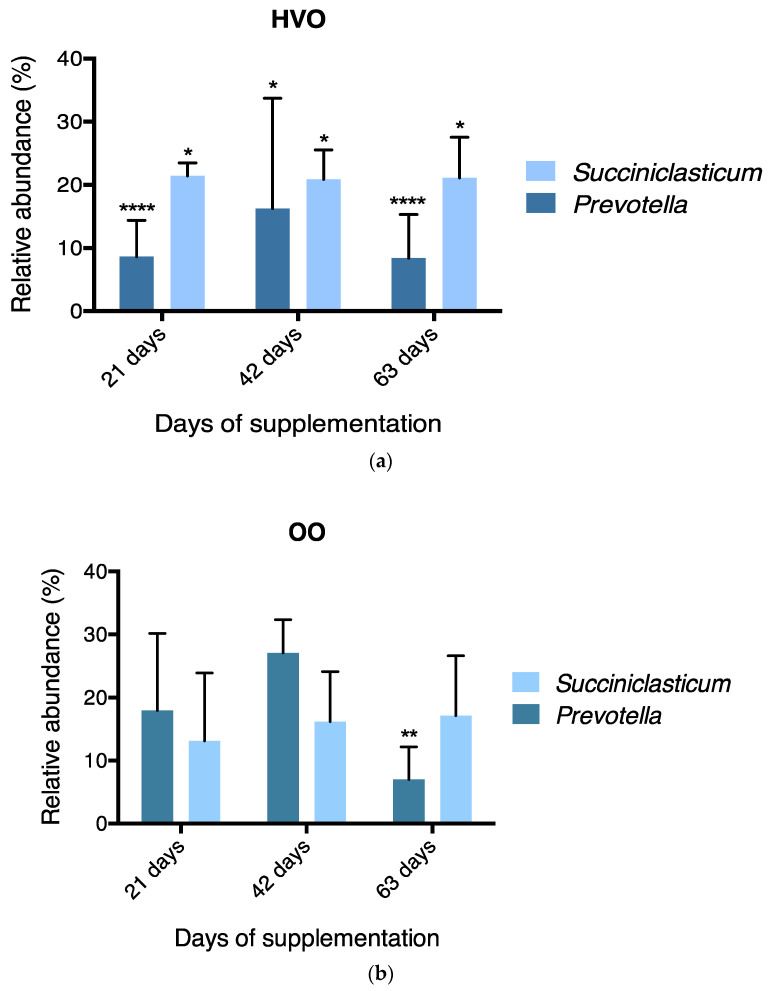
Effect of dietary treatment and supplementation time on relative abundance of the two dominant genera in rumen microbiota, (**a**) HVO and (**b**) OO. HVO, supplemented with 3% DM hydrogenated vegetable oil; OO, supplemented with 3% DM olive oil. *p*-values were obtained with Dunnett’s multiple comparisons test. * *p* ≤ 0.05; ** *p* ≤ 0.01; **** *p* ≤ 0.0001.

At the phylum level, 17 phyla were identified in the ruminal samples irrespective of diet (Figure 1a), with phyla Firmicutes (71.5%), followed by Bacteroidetes (26.2%) and Actinobacteria (1%), accounting for 98.7% of the phyla members. Less abundant phyla averaging a relative abundance of 0.5% or less were grouped as ‘Others’. Table S1 compares the mean relative abundance (%) at different time points of the most prevalent phylum between control, HVO and OO, showing no significant effects between treatments. With regard to temporal changes at the phylum level, the results show that with HVO the relative abundance of Bacteroidetes decreased (*p* ≤ 0.001) on day 21 and 63 with HVO addition, whereas Firmicutes increased (*p* ≤ 0.001) on the same days (Dunnett’s test, Figure 2a). With regard to OO supplementation, Bacteroidetes decreased (*p* ≤ 0.001) and Firmicutes increased (*p* ≤ 0.001) on day 63 (Dunnett’s test, Figure 2b). Eighty bacterial families were identified within rumen samples, where 23 of those members accounted for a relative abundance of ≥ 1% (Figure 1b). The main family groups were *Ruminococcaceae* (22.8%), *Lachnospiraceae* (21.3%), *Prevotellaceae* (19.7%) and *Acidaminococcaceae* (13.5%). Family groups did not change between treatments (Table S2). Mean relative abundances of the most prevalent genera are shown in Table S3. However, finer genus-level data showed a relative abundance of ≥ 1% for 70 taxa (Figure 1c). *Prevotella* (*Bacteroidetes/Prevotellaceae*) and *Succiniclasticum* (*Firmicutes/Acidaminococcaceae*) were dominant, with mean relative abundance of 19.4 and 16.6% respectively. The effect of lipid supplementation on relative abundance of these predominant genera is shown in Figure 3. *Prevotella* was reduced (*p* ≤ 0.05) in both HVO (days 21, 42 and 63, Figure 3a) and OO (day 63, Figure 3b) diets, whereas *Succiniclasticum* only increased (*p* ≤ 0.05) with HVO after 21, 42 and 63 days of supplementation.

**Figure 4:**
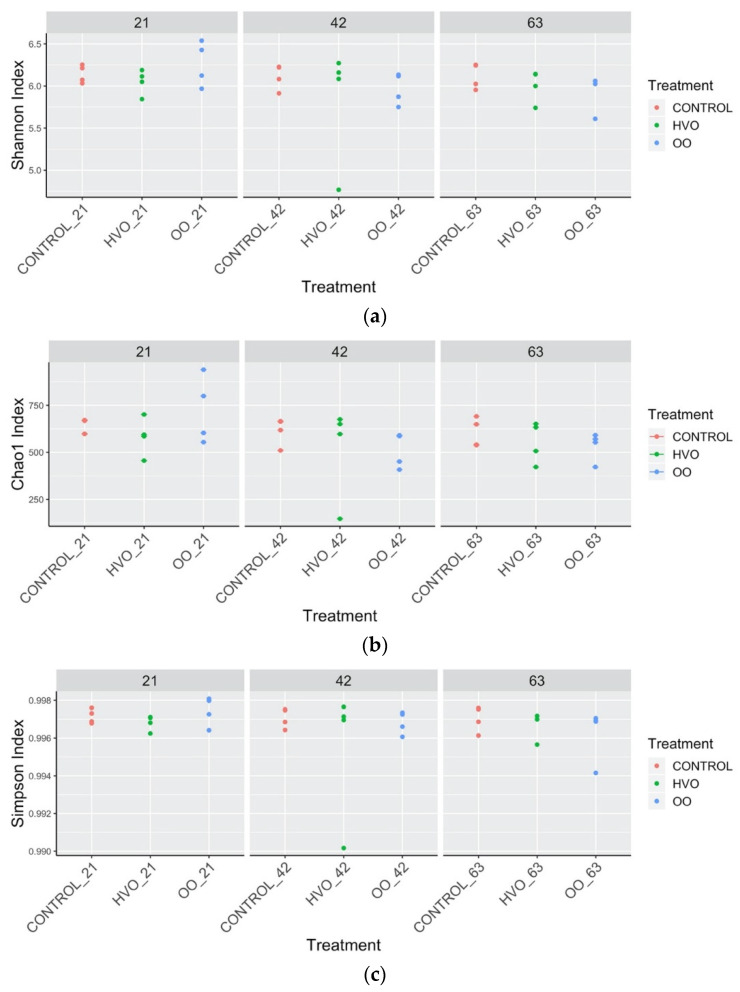
Ruminal microbial richness and diversity with lipid supplementation throughout the experimental periods. Bacterial diversity estimated by (**a**) Shannon Index and (**b**) Simpson Index, and bacterial richness estimated by the (**c**) Chao1 value. Control, no fat supplement; HVO, supplemented with 3% DM hydrogenated vegetable oil; OO, supplemented with 3% DM olive oil. Supplementation periods of 21, 42 and 63 days.

**Figure 5:**
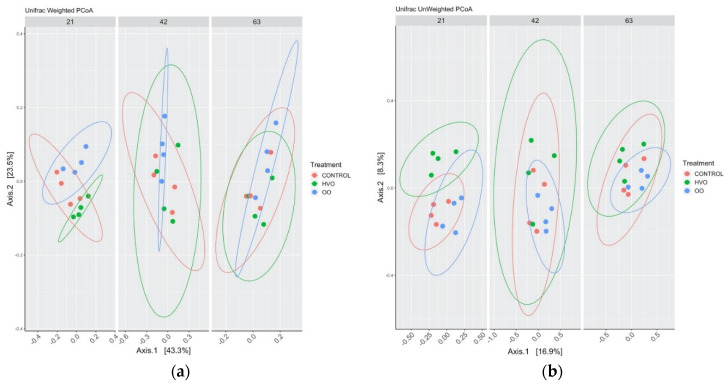
Principal coordinate analysis (PCoA) of bacterial community structures of the ruminal microbiota in the control (red points), HVO (green points) and OO (blue points), constructed using the (**a**) weighted UniFrac and (**b**) unweighted Unifrac method. Control, no fat supplement; HVO, supplemented with 3% DM hydrogenated vegetable oil; OO, supplemented with 3% DM olive oil. Supplementation periods of 21, 42 and 63 days.

Alpha diversity was not altered by diet. Shannon (Figure 4a), Chao1 (Figure 4b) and Simpson (Figure 4c) diversity indices were not significantly different between each dietary treatment. Beta diversity between the samples at four different time points during lipid supplementation was evaluated. In both the weighted (Figure 5a) and unweighted (Figure 5b) UniFrac distances, the closer positions of the samples in the Principal Component Analysis (PCoA), indicate similar microbial composition between them, showing no major differentiation among rumen bacterial communities following feeding with all diets. PERMANOVA confirmed the absence of significant differences (*p* > 0.05) in the composition of the rumen microbiota in weighted and unweighted results. Detailed information can be found in Tables S4 and S5.

### 3.3. Prediction of Function

**Figure 6:**
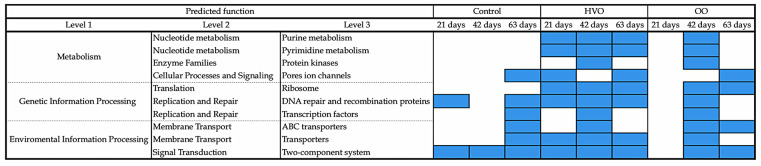
Common predicted functions with significant variations in control, hydrogenated vegetable oil (HVO) and olive oil (OO) treatments. Control, no fat supplement; HVO, supplemented with 3% DM hydrogenated vegetable oil; OO, supplemented with 3% DM olive oil. Supplementation periods of 21, 42 and 63 days. Results were declared significant at *p* < 0.01.

Predicted functional features of the rumen bacterial community obtained using CowPI showed 255 gene families identified in ruminal samples. Functional pathways that were examined by dietary treatments were grouped under five general categories: (1) genes and proteins (37.9%), (2) metabolism (34.5%), (3) genetic information processing (10.3%), (4) unclassified (10.3%) and (5) environmental information processing (6.9%). In particular, 10 functions showed significant differences among the dietary treatments (Figure 6). Those functions were related to ABC transporters, DNA repair and recombination proteins, pore ion channels, protein kinases, purine metabolism, pyrimidine metabolism, ribosome, transcription factors, transporters and the two-component system. Metabolic pathways with statistical variations were observed mainly in the diet supplemented with HVO and at day 42 of OO supplementation. The most interesting included: purine metabolism, pyrimidine metabolism and ribosome. Details on predicted functions from individual treatments can be found in the Supplementary Materials (Tables S6–S8). Raw pathways obtained from CowPI data were categorized according to the KEGG database (Table S9).

## 4. Discussion

The gastrointestinal microbiome in bovines performs several physiological functions that are lacking in the host, and therefore can be considered essential to their life [1,2]. The rumen microbiome is central to ensure the productivity and health of ruminants; therefore, any supplements to diets have to be verified to ensure that there are no detrimental effects [25,26,27]. Consequently, the aims of this study were to investigate, through an Illumina Miseq sequencing approach, the effect of supplementing HVO and OO at 3% DM on the rumen bacterial community of dairy cows over a relatively long timescale of 63 days (alongside monitoring fermentation). Inclusion of these oil by-products can supply dietary energy as well as increasing dairy products’ contents of beneficial FA for human health. Similar studies have used OO by-products and analyzed the effects on the rumen microbiome, for example in sheep fed with OO pomace over 28 days [14] and an in *vitro* study [26] where OO, sunflower oil and linseed oil was tested at 6% DM inclusion. In this study, lipid supplementation was included in the diet at 3% DM as rumen microbes are generally intolerant to high levels of fat in the diet [28]. This approach was expected to produce some changes in milk FA profiles without detrimental effects on overall productive traits, as has been reported in detail [12,29,30,31]. This is the first *in vivo* study to characterize the effect of two different lipid sources, HVO (as saturated fatty acid source) and OO (as unsaturated fatty acid source), on the composition of the rumen bacterial community in dairy cows over a relatively long-term period of 63 days. 

Lipid supplementation did not affect ruminal pH and results were within the normal biological range [32], with a minimum of 6.98 and a maximum of 7.11 pH, which could indicate that cellulolytic processes of fiber digestion were unaffected, and microbes adapted to the diet. Nur Atikah et al. [33] suggested that an adequate roughage supply in the diet could reduce the negative effect of dietary oil on rumen fermentation because the fiber fraction creates a supporting environment for rumen microbes to hydrolyze the dietary oils. 

As expected, NH_3_-N was not affected by treatments. Reductions in NH_3_-N concentration have been associated with defaunation or inhibition of the hyper-ammonia-producing bacteria [34]. It is well known that NH_3_-N is an intermediate product of feed protein, non-protein nitrogen degradation and microbial protein synthesis, and it is mainly affected by feed protein degradation, rumen wall absorption, microorganism utilization and rumen chyme outflow rate [35]. 

The lack of differences in total VFA concentration and proportions of individual VFA between treatments was similar to that reported by Benchaar et al. [36], who did not find differences in rumen pH and total VFA concentrations in dairy cows fed with 3% DM linseed oil. Similarly, in another study where cows were supplemented with either soybean oil or hydrogenated vegetable oil at 2.7% DM, rumen pH, total VFA and individual proportions of VFA were not affected [21]. Although some individual VFA were decreased or increased through the experimental periods, the magnitude of changes is not expected to be of biological significance, and this is also supported by the animal’s performance data, where milk production and components were not changed [13]. In general, the observed differences in ruminal parameters were not significant, which could suggest that the level of lipid supplementation was not high enough to affect ruminal metabolism, and was likely a consequence of the resilience and redundancy of the rumen microbiome [37]. 

Consistent with previous reports [38,39,40], Bacteroidetes and Firmicutes were the most abundant phyla, accounting in the microbiome data for 97.7% of the bacteria in the rumen samples. Both these phyla play a major role in the degradation of fiber and polysaccharides [41] and are therefore part of the core microbiome of cattle, with genera within these phyla, namely *Ruminococcus*, *Butyrivibrio* (Firmicutes) and *Prevotella* (Bacteroidetes), being critical for energy harvesting [42]. In this study, Bacteroidetes/Firmicutes showed compensatory changes in their relative abundance. Loor et al. [43] proposed that Bacteroidetes are dominant in the rumen from 6 weeks of life, and this dominance is independent of sampling time [44] and diet [45,46]. In addition, Pitta et al. [47] established that irrespective of the source of oil supplements used, higher concentrations of PUFA could be detrimental to Bacteroidetes. Matthews et al. [1] also proposed that higher percentages of Firmicutes compensated for the lower abundances of Bacteroidetes due to the redundancy within these phyla, which could explain the data obtained in this study. 

Shannon, Chao1 and Simpson diversity indices showed no significant effects of dietary treatment, which is in disagreement with Bayat et al. [48], who proposed that lipid supplements altered the diversity of rumen microbial communities and relative abundances of some common taxa, as opposed to having a global response. However, Huws et al. [49] and Pitta et al. [47] found that the number of bacterial populations (species richness) and their distribution (diversity) changed under different oil supplements, likely due to the different oils and levels used in those studies. In this study and at the oil levels used, the lack of differences observed between dietary treatments may simply indicate that the induced changes in microbial communities lie at a finer genus-level resolution [50]. 

Genus-level data showed that *Prevotella* and *Succiniclasticum* were dominant across all the samples, control samples and dietary treatments. This result is in concordance with Pitta et al. [51] and Wirth et al. [52], who reported that *Prevotella* is the most predominant ruminal genus, accounting for 42 to 60% of the bacterial 16S rRNA gene sequences, and is more abundant in liquid fractions of ruminal samples [38,53]. We observed differences in relative abundance of predominant genera over time and dependent on the lipid source. Relative abundance of *Prevotella* decreased following 63 days of both HVO and OO dietary supplementation. Contrarily, *Succiniclasticum* increased in abundance following both HVO and OO dietary supplementation throughout the study. Bi et al. [54] found that the relative abundances of the genera *Succiniclasticum* significantly increased with increasing dietary energy levels, which could explain our results as lipid supplementation (saturated or unsaturated FA source) increases energy content in the dietary treatments. It has been established that dietary composition plays an important role in determining both the community structure and the metabolic function of the rumen microbiota [55]; for example, Huang et al. [56] found that the relative abundance of *Prevotella* and *Succiniclasticum* were positively correlated with several pathways, such as protein metabolism, carbohydrate metabolism and lipid metabolism, among others.

Lastly, CowPI was used to study bacterial gene functions in rumen, and the results showed that predicted pathways were modulated in rumen according to the diet. All of these pathways are essential for bacterial growth and overall animal performance, as they are related to metabolism, genetics and environmental information processing [46]. Metabolic pathways with statistical variations were observed mainly at day 42 of supplementation, and they included genes that could reveal damage in DNA, which could be associated with the effect of lipid supplementations; for example, some increase in redox reactions or pathways. This an interesting point to address in future experiments. 

Although OO was a dietary lipid, a source rich in unsaturated FA characterized by 74/100 g of C18:1 *cis*-9, and HVO was a source rich in saturated FA, with 58/100 g of C16:0 and 40/100 g of C18:0 [13], rumen microbiome changes to these dietary supplements were marginal. At a molecular level, we also previously observed mild effects on the expression of lipid-related genes in subcutaneous adipose tissue [57] and milk somatic cells [58], but achieved improvements in PUFA C18:1 *cis*-9 and C18:3 *cis*-9, *cis*-12, *cis*-15 in milk content of milk following OO dietary supplementation. Taken together, the responses observed from both OO and HVO dietary supplementation suggest that long-term supplementation at 3% DM inclusion is an effective source of energy and can improve the PUFA content of milk (OO) whilst no detrimental effect on the rumen microbiome occurs (above 5% DM inclusion could be detrimental [59]). 

## 5. Conclusions

This study provides a comprehensive evaluation of long-term supplementation of saturated (hydrogenated palm oil) and monounsaturated (unrefined olive oil) fatty acids sources on rumen bacteria using a sequencing approach. Overall, 3% DM lipid supplementation of either OO or HVO, over 63 days (9 weeks), can be used in dairy cow diets without major impacts on global bacterial community structure.

## Figures and Tables

**Table 1:** Effect of hydrogenated vegetable (HVO) and olive (OO) oil on ruminal fermentation parameters.

Rumen Parameter	Treatment					*p*-Value	
	Control	HVO	OO	SEM	Diet (D)	Time (T)	D × T
pH	6.98	7.10	6.97	0.07	0.157	0.901	0.432
NH_3_-N, mg/dL	5.42	4.39	4.76	1.21	0.217	0.064	0.291
Total VFA (mmol/L)	96.1	73.0	82.5	19.5	0.069	0.001	0.605
Molar proportion (mol/100 mol)							
Acetate	66.3	65.4	64.4	3.69	0.559	0.021	0.300
Propionate	18.6	19.3	20.0	4.03	0.746	0.001	0.184
Butyrate	9.62	9.04	8.85	1.26	0.412	0.024	0.234
Valerate	1.66	1.84	2.00	0.52	0.376	0.104	0.692
Isovalerate	1.59	2.16	1.71	0.63	0.164	0.279	0.480
Isobutyric	1.97	2.05	2.54	0.83	0.310	0.013	0.301

Control, no fat supplement; HVO, supplemented with 3% DM hydrogenated vegetable oil; OO, supplemented with 3% DM olive oil; SEM: standard error of the mean; results were declared significant at *p* < 0.05.

## Data Availability

The data presented in this study are available on request from the corresponding author.

## Supplementary Materials

The following are available online at https://www.mdpi.com/article/10.3390/microorganisms9061121/s1, Table S1: Most Prevalent Taxa: Phylum level; Table S2: Most Prevalent Taxa: Family level; Table S3. Most Prevalent Taxa: Genus level; Table S4. PERMANOVA (Weighted) results; Table S5. PERMANOVA (Unweighted) results; Table S6. Predicted Functions in Control Diet; Table S7. Predicted Functions in OO Diet; Table S8. Predicted Functions in HVO Diet; Table S9. KEGG classification of functional gene categories.

## References

[1] Matthews C., Crispie F., Lewis E., Reid M., O’Toole P.W., Cotter P.D. (2019). The rumen microbiome: A crucial consideration when optimising milk and meat production and nitrogen utilisation efficiency. Gut Microbes.

[2] Sirohi S., Singh N., Dagar S., Puniya A. (2012). Molecular tools for deciphering the microbial community structure and diversity in rumen ecosystem. Appl. Microbiol. Biotechnol..

[3] Huws S.A., Creevey C.J., Oyama L.B., Mizrahi I., Denman S.E., Popova M., Muñoz-Tamayo R., Forano E., Waters S.M., Hess M. (2018). Addressing global ruminant agricultural challenges through understanding the rumen microbiome: Past, present, and future. Front. Microbiol..

[4] Wang M., Wang R., Xie T.Y., Janssen P.H., Zhao Sun X., Beauchemin K.A., Tan Z.L., Gao M. (2016). Shifts in Rumen Fermentation and Microbiota Are Associated with Dissolved Ruminal Hydrogen Concentrations in Lactating Dairy Cows Fed Different Types of Carbohydrates. J. Nutr..

[5] Maia M.R., Chaudhary L.C., Figueres L., Wallace R.J. (2007). Metabolism of polyunsaturated fatty acids and their toxicity to the microflora of the rumen. Antonie Van Leeuwenhoek.

[6] Maia M.R., Chaudhary L.C., Bestwick C.S., Richardson A.J., McKain N., Larson T.R. (2010). Toxicity of unsaturated fatty acids to the biohydrogenating ruminal bacterium, Butyrivibrio fibrisolvens. BMC Microbiol..

[7] Toral P.G., Monahan F.J., Hervas G., Frutos P., Moloney A.P. (2018). Review: Modulating ruminal lipid metabolism to improve the fatty acid composition of meat and milk. challenges and opportunities. Animal.

[8] Vargas-Bello-Pérez E., Garnsworthy P.C. (2013). Trans fatty acids and their role in the milk of dairy cows. Int. J. Agric. Nat. Resour..

[9] Fuke G., Laerte Nornberg J. (2017). Systematic evaluation on the effectiveness of conjugated linoleic acid in human health. Crit. Rev. Food Sci. Nutr..

[10] Vargas-Bello-Pérez E., Íñiguez-González G., Fehrmann-Cartes K., Toro-Mujica P., Garnsworthy P.C. (2015). Influence of fish oil alone or in combination with hydrogenated palm oil on sensory characteristics and fatty acid composition of bovine cheese. Anim. Feed Sci. Technol..

[11] Welter K.C., Marlon de Magalhães Rodrigues Martins C., Soligo Vizeu de Palma A., Martinson Martins M., Roqueto dos Reis B., Unglaube Schmidt B.L., Saran Netto A. (2016). Canola Oil in Lactating Dairy Cow Diets Reduces Milk Saturated Fatty Acids and Improves Its Omega-3 and Oleic Fatty Acid Content. PLoS ONE.

[12] Vargas-Bello-Pérez E., Geldsetzer-Mendoza C., Morales S.M., Toro-Mujica P., Fellenberg M.A., Ibáñez R.A. (2018). Effect of olive oil in dairy cow diets on the fatty acid profile and sensory characteristics of cheese. Int. Dairy J..

[13] Vargas-Bello-Pérez E., Cancino-Padilla N., Geldsetzer-Mendoza C., Vyhmeister S., Morales M.S., Leskinen H. (2019). Effect of Feeding Cows with Unsaturated Fatty Acid Sources on Milk Production, Milk Composition, Milk Fatty Acid Profile, and Physicochemical and Sensory Characteristics of Ice Cream. Animals.

[14] Mannelli F., Cappucci A., Pini F., Pastorelli R., Decorosi F., Giovannetti L. (2018). Effect of different types of olive oil pomace dietary supplementation on the rumen microbial community profile in Comisana ewes. Sci. Rep..

[15] Vargas-Bello-Pérez E., Cancino-Padilla N., Romero J., Garnsworthy P.C. (2016). Quantitative analysis of ruminal bacterial populations involved in lipid metabolism in dairy cows fed different vegetable oils. Animal.

[16] Snelling T.J., Auffret M.D., Duthie C., Stewart R.D., Watson M., Dewhurst R.J. (2019). Temporal stability of the rumen microbiota in beef cattle, and response to diet and supplements. Anim. Microbiome.

[17] Abecia L., Jiménez E., Martínez-Fernandez G., Martín-García A.I., Ramos-Morales E., Pinloche E. (2017). Natural and artificial feeding management before weaning promote different rumen microbial colonization but not differences in gene expression levels at the rumen epithelium of newborn goats. PLoS ONE.

[18] Wildman E.E., Jones G.M., Wagner P.E., Boman R.L., Troutt H.F., Lesch T.N. (1982). A dairy cow body condition scoring system and its relationship to selected production characteristics. J. Dairy Sci..

[19] NRC (2001). Nutrient Requirements of Dairy Cattle.

[20] Geishauser T., Linhart N., Neidl A., Reimann A. (2012). Factors associated with ruminal pH at herd level. J. Dairy Sci..

[21] Caporaso J.G., Lauber C.L., Walters W.A., Berg-Lyons D., Lozupone C.A., Turnbaugh P.J., Fierer N., Knight R. (2011). Global patterns of 16S rRNA diversity at a depth of millions of sequences per sample. Proc. Natl. Acad. Sci. USA.

[22] Lokesh J., Kiron V. (2016). Transition from freshwater to seawater reshapes the skin-associated microbiota of Atlantic salmon. Sci. Rep..

[23] McMurdie P.J., Holmes S. (2013). Phyloseq: An R Package for Reproducible Interactive Analysis and Graphics of Microbiome Census Data. PLoS ONE.

[24] Wilkinson T.J., Huws S.A., Edwards J.E., Kingston-Smith A.H., Siu-Ting K., Hughes M., Rubino F., Friedersdorff M., Creevey C.J. (2018). CowPI: A rumen microbiome focussed version of the PICRUSt functional inference software. Front. Microbiol..

[25] Wanapat M., Cherdthong A., Phesatcha K., Kang S. (2015). Dietary sources and their effects on animal production and environmental sustainability. Anim. Nutr..

[26] Vargas J.E., Andrés A., López-Ferreras L., Snelling T.J., Yáñez-Ruíz D.R., García-Estrada C. (2020). Dietary supplemental plant oils reduce methanogenesis from anaerobic microbial fermentation in the rumen. Sci. Rep..

[27] Szumacher-Strabel M., Potkański A., Kowalczyk J., Cieślak A., Czauderna M., Gubała A. (2002). The influence of supplemental fat on rumen volatile fatty acid profile, ammonia and pH levels in sheep fed a standard diet. J. Anim. Feed Sci..

[28] Altenhofer C., Spornraft M., Kienberger H., Rychlik M., Herrmann J., Meyer H.H.D. (2014). Effects of rapeseed and soybean oil dietary supplementation on bovine fat metabolism, fatty acid composition and cholesterol levels in milk. J. Dairy Res..

[29] Chamberlain M.B., DePeters E.J. (2017). Impacts of feeding lipid supplements high in palmitic acid or stearic acid on performance of lactating dairy cows. J. Appl. Anim. Res..

[30] Bougouin A., Martin C., Doreau M., Ferlay A. (2019). Effects of starch-rich or lipid-supplemented diets that induce milk fat depression on rumen biohydrogenation of fatty acids and methanogenesis in lactating dairy cows. Animal.

[31] Grünberg W., Constable P.D. (2009). Function and Dysfunction of the Ruminant Forestomach. Current Veterinary Therapy.

[32] Nur Atikah I., Alimon A.R., Yaakub H., Abdullah N., Jahromi M.F., Ivan M., Samsudin A.A. (2018). Profiling of rumen fermentation, microbial population and digestibility in goats fed with dietary oils containing different fatty acids. BMC Vet. Res..

[33] Bach A., Calsamiglia S., Stern M. (2005). Nitrogen metabolism in the rumen. J. Dairy Sci..

[34] Tong J., Zhang H., Yang D., Zhang Y., Xiong Y., Jiang L. (2018). Illumina sequencing analysis of the ruminal microbiota in high-yield and low-yield lactating dairy cows. PLoS ONE.

[35] Benchaar C., Romero-Pérez G.A., Chouinard P.Y., Hassanat F., Eugene M., Petit H.V., Côrtes C. (2012). Supplementation of increasing amounts of linseed oil to dairy cows fed total mixed rations: Effects on digestion, ruminal fermentation characteristics, protozoal populations, and milk fatty acid composition. J. Dairy Sci..

[36] Vargas-Bello-Pérez E., Geldsetzer-Mendoza C., Cancino-Padilla N., Morales M.S., Leskinen H., Garnsworthy P.C. (2020). Effects of Dietary Vegetable Oils on Mammary Lipid-Related Genes in Holstein Dairy Cows. Animals.

[37] Lyons T., Boland T., Storey S., Doyle E. (2017). Linseed oil supplementation of lambs’ diet in early life leads to persistent changes in rumen microbiome structure. Front. Microbiol..

[38] Kim M., Morrison M., Yu Z. (2011). Status of the phylogenetic diversity census of ruminal microbiomes. FEMS Microbiol. Ecol..

[39] Jewell K.A., McCormick C.A., Odt C.L., Weimer P.J., Suen G. (2015). Ruminal Bacterial Community Composition in Dairy Cows Is Dynamic over the Course of Two Lactations and Correlates with Feed Efficiency. Appl. Environ. Microbiol..

[40] Zhu Z., Kristensen L., Difford G.F., Poulsen M., Noel S.J., Al-Soud W.A., Sørensen S.J., Lassen J., Løvendahl P., Højberg O. (2018). Changes in rumen bacterial and archaeal communities over the transition period in primiparous Holstein dairy cows. J. Dairy Sci..

[41] Cheng Y., Wang Y., Li Y., Zhang Y., Liu T., Wang Y., Sharpton T.J., Zhu W. (2017). Progressive colonization of bacteria and degradation of rice straw in the rumen by Illumina sequencing. Front. Microbiol..

[42] Weimer P.J. (2015). Redundancy, resilience, and host specificity of the ruminal microbiota: Implications for engineering improved ruminal fermentations. Front. Microbiol..

[43] Loor J.J., Elolimy A.A., McCann J.C. (2016). Dietary impacts on rumen microbiota in beef and dairy production. Anim. Front..

[44] Cremonesi P., Conte G., Severgnini M., Turri F., Monni A., Capra E., Rapetti L., Colombini S., Chessa S., Battelli G. (2018). Evaluation of the effects of different diets on microbiome diversity and fatty acid composition of rumen liquor in dairy goat. Animal.

[45] McCann J.C., Wickersham T.A., Loor J.J. (2014). High-throughput methods redefine the rumen microbiome and its relationship with nutrition and metabolism. Bioinform. Biol. Insights.

[46] Paz H.A., Hales K.E., Wells J.E., Kuehn L.A., Freetly H.C., Berry E.D., Flythe M.D., Spangler M.L., Fernando S.C. (2018). Rumen bacterial community structure impacts feed efficiency in beef cattle. J. Anim. Sci..

[47] Pitta D.W., Indugu N., Vecchiarelli B., Rico D.E., Harvatine K.J. (2018). Alterations in ruminal bacterial populations at induction and recovery from diet-induced milk fat depression in dairy cows. J. Dairy Sci..

[48] Bayat A.R., Tapio I., Vilkki J., Shingfield K.J., Leskinen H. (2017). Plant oil supplements reduce methane emissions and improve milk fatty acid composition in dairy cows fed grass silage-based diets without affecting milk yield. J. Dairy Sci..

[49] Huws S.A., Kim E.J., Cameron S.J.S., Girdwood S.E., Davies L., Tweed J., Vallin H., Scollan N.D. (2015). Characterization of the rumen lipidome and microbiome of steers fed a diet supplemented with flax and echium oil. Microb. Biotechnol..

[50] Myer P.R., Smith T.P.L., Wells J.E., Kuehn L.A., Freetly H.C. (2015). Rumen Microbiome from Steers Differing in Feed Efficiency. PLoS ONE.

[51] Pitta D.W., Pinchak E., Dowd S.E., Osterstock J., Gontcharova V., Youn E., Dorton K., Yoon I., Min B.R., Fulford J.D. (2010). Rumen bacterial diversity dynamics associated with changing from bermudagrass hay to grazed winter wheat diets. Microb. Ecol..

[52] Wirth R., Kádár G., Kakuk B., Maróti G., Bagi Z., Szilágyi A., Rákhely G., Horváth J., Kovács K.L. (2018). The planktonic core microbiome and core functions in the cattle rumen by next generation sequencing. Front. Microbiol..

[53] Koringa P.G., Thakkar J.R., Pandit R.J., Hinsu A.T., Parekh M.J., Shah R.K., Jakhesara S.J., Joshi C.G. (2019). Metagenomic characterisation of ruminal bacterial diversity in buffaloes from birth to adulthood using 16S rRNA gene amplicon sequencing. Funct. Integr. Genom..

[54] Bi Y., Zeng S., Zhang R., Diao Q., Tu Y. (2018). Effects of dietary energy levels on rumen bacterial community composition in Holstein heifers under the same forage to concentrate ratio condition. BMC Microbiol..

[55] Ren H., Su X., Bai H., Yang Y., Wang H., Dan Z., Lu J., Wu S., Cai C., Cao Y. (2019). Specific enrichment of microbes and increased ruminal propionate production: The potential mechanism underlying the high energy efficiency of Holstein heifers fed steam-flaked corn. AMB Expr..

[56] Huang S., Ji S., Yan H., Hao Y., Zhang J., Wang Y., Cao Z., Li S. (2020). The day-to-day stability of the ruminal and fecal microbiota in lactating dairy cows. Microbiologyopen.

[57] Vargas-Bello-Pérez E., Bionaz M., Sciarresi-Arechabala P., Cancino-Padilla N., Morales M.S., Romero J., Leskinen H., Garnsworthy P.C., Loor J.J. (2019). Long-Term Effects of Dietary Olive Oil and Hydrogenated Vegetable Oil on Expression of Lipogenic Genes in Subcutaneous Adipose Tissue of Dairy Cows. Vet. Sci..

[58] Vargas-Bello-Pérez E., Cancino-Padilla N., Geldsetzer-Mendoza C., Morales M.S., Leskinen H., Garnsworthy P.C., Loor J.J., Romero J. (2020). Effects of dietary polyunsaturated fatty acid sources on expression of lipid-related genes in bovine milk somatic cells. Sci Rep..

[59] Bionaz M., Vargas-Bello-Pérez E., Busato S. (2020). Advances in fatty acids nutrition in dairy cows: From gut to cells and effects on performance. J. Anim. Sci. Biotechnol..

