# Supplementary material for: Long-Term Effects of Dietary Supplementation with Olive Oil and Hydrogenated Vegetable Oil on the Rumen Microbiome of Dairy Cows
Source: Microorganisms. 2021 May 22;9(6):1121. doi: 10.3390/microorganisms9061121 (PMC8224598; doi:10.3390/microorganisms9061121)
Supplement: Supplementary file 1 [file microorganisms-09-01121-s001.zip › microorganisms-1230005-supplementary.pdf]

Long-term effects of dietary supplementation with olive oil and hydrogenated vegetable oil on the rumen microbiome of dairy cows

Nathaly Cancino-Padilla[1], Natalia Catalán[2], Karen Siu-Ting[3], Christopher J. Creevey[3], Sharon A. Huws[3], Jaime Romero[2] and Einar Vargas-Bello-Pérez[4, 1*]

[1]Departamento de Ciencias Animales, Facultad de Agronomía e Ingeniería Forestal, Pontificia Universidad Católica de Chile, Avda. Vicuña Mackenna 4860, Santiago 6904411, Chile; nathaly.cancino@gmail.com

[2]Laboratorio de Biotecnología de Alimentos, Unidad de Alimentos, Instituto de Nutrición y Tecnología de los Alimentos (INTA), Universidad de Chile, El Líbano 5524, Macul, Santiago 7830490, Chile; nataliabcatalant@gmail.com

[3]Institute for Global Food Security, School of Biological Sciences, Queen's University of Belfast, 19 Chlorine Gardens, Belfast BT9 5DL, UK; agalychnica@gmail.com (K.S.-T.); chris.creevey@qub.ac.uk (C.J.C.); s.huws@qub.ac.uk (S.A.H.)

[4]Department of Veterinary and Animal Sciences, Faculty of Health and Medical Sciences, University of Copenhagen, Grønnegårdsvej 3, DK-1870 Frederiksberg C, Denmark

*Correspondence: jromero@inta.uchile.cl (J.R.); evargasb@sund.ku.dk (E.V.-B.-P.)

Supplementary material

Table S1. Most Prevalent Taxa: Phylum level.
Mean relative abundance (%) at different time point of the most prevalent phylum from cows fed with control, HVO and OO treatments

Phylum	Treatments			SEM[4]	*P*-value
	Control[1]	HVO[2]	OO[3]		
21 days					
Firmicutes	60.16	82.75	72.83	6.54	0.10
Bacteroidetes	37.84	15.03	23.78	6.64	0.10
Actinobacteria	0.80	0.76	2.20	0.47	0.13
Proteobacteria	0.29	0.40	0.52	0.07	0.25
Others	0.91	1.06	0.67	0.12	0.37
42 days					
Firmicutes	65.97	74.68	64.82	3.11	0.63
Bacteroidetes	31.60	22.25	32.05	3.19	0.54
Actinobacteria	1.02	1.44	1.71	0.20	0.82
Proteobacteria	0.75	0.99	1.03	0.09	0.56
Others	0.66	0.63	0.39	0.08	0.54
63 days					
Firmicutes	81.19	84.92	87.42	1.81	0.59
Bacteroidetes	16.39	12.61	9.612	1.96	0.51
Actinobacteria	1.56	1.15	2.19	0.30	0.35
Proteobacteria	0.29	0.35	0.44	0.05	0.35
Others	0.57	0.97	0.34	0.18	0.07

[1]Control, no fat supplement; [2]HVO, supplemented with 3% DM hydrogenated vegetable oil; [3]OO, supplemented with 3% DM olive oil; [4]SEM: standard error of the mean and values are LSM and pooled SEM; [5]Others, less abundant Phylum (relative abundance ≤ 0.5%). Data were analyzed by one-way ANOVA and means were compared by Kruskal-Wallis test. Results were declared significant at $P<0.05$.

Table S2. Most Prevalent Taxa: Family level.

Mean relative abundance (%) at different time point of the most prevalent families from cows fed with control, HVO and OO treatments

Family	Treatments			SEM[4]	*P*-value
	Control[1]	HVO[2]	OO[3]		
21 days					
Ruminococcaceae	17.42	29.60	22.84	3.52	0.06
Lachnospiraceae	12.74	19.05	23.09	3.01	0.15
Prevotellaceae	30.30	9.78	18.54	5.95	0.12
Acidaminococcaceae	16.24	18.41	10.41	2.39	0.40
Others	23.29	23.17	25.13	0.63	0.75
42 days					
Ruminococcaceae	17.59	21.90	16.86	1.57	0.63
Lachnospiraceae	19.05	22.35	21.39	0.98	0.84
Prevotellaceae	26.80	18.15	28.66	3.24	0.54
Acidaminococcaceae	17.01	16.99	13.14	1.29	0.63
Others	19.55	20.62	19.95	0.31	0.99
63 days					
Ruminococcaceae	21.15	25.31	25.52	1.42	0.58
Lachnospiraceae	29.12	23.96	35.39	3.30	0.15
Prevotellaceae	12.37	8.93	7.58	1.43	0.54
Acidaminococcaceae	14.70	16.88	12.69	1.21	0.71
Others	22.67	24.92	18.82	1.78	0.05

[1]Control, no fat supplement; [2]HVO, supplemented with 3% DM hydrogenated vegetable oil; [3]OO, supplemented with 3% DM olive oil; [4]SEM: standard error of the mean standard error of the mean and values are LSM and pooled SEM.[5]'Others' refers to the less abundant Phylum (relative abundance ≤ 0.5%). Data were analyzed by one-way ANOVA and means were compared by Kruskal-Wallis test. Results were declared significant at P<0.05.

Table S3. Most Prevalent Taxa: Genus level.

Mean relative abundance (%) at different time point of the most prevalent genus from cows fed with control, HVO and OO treatments

Genus	Treatments [1]Control	[2]HVO	[3]OO	SEM	*P*- value
21 days					
Prevotella_1	29.15	8.63	17.97	5.93	0.20
Succiniclasticum	19.13	21.44	13.12	2.48	0.45
Ruminococcaceae_NK4A214_group	9.16	13.65	11.65	1.30	0.20
Christensenellaceae_R-7_group	7.83	10.46	6.57	1.15	0.27
Ruminococcaceae_UCG-014	5.31	9.26	6.19	1.20	0.25
Lachnospiraceae_NK3A20_group	3.03	4.86	6.12	0.89	0.33
Others	0.262	0.315	0.382	0.035	0.001
42 days					
Prevotella_1	24.95	16.28	27.10	3.31	0.54
Succiniclasticum	21.07	20.87	16.23	1.58	0.54
Ruminococcaceae_NK4A214_group	7.89	10.11	8.28	0.69	0.77
Christensenellaceae_R-7_group	7.25	8.00	4.95	0.92	0.71
Ruminococcaceae_UCG-014	4.08	8.55	4.30	1.46	0.45
Lachnospiraceae_NK3A20_group	4.61	6.28	5.25	0.49	0.91
Others	0.298	0.298	0.338	0.013	0.82
63 days					
Prevotella_1	12.09	8.42	6.99	1.52	0.54
Succiniclasticum	18.74	21.09	17.14	1.15	0.59
Ruminococcaceae_NK4A214_group	11.35	12.04	15.74	1.36	0.51
Christensenellaceae_R-7_group	10.95	12.12	6.43	1.73	0.09
Ruminococcaceae_UCG-014	4.66	4.75	5.41	0.24	0.91
Lachnospiraceae_NK3A20_group	7.82	6.25	11.56	1.58	0.23
Others	0.343	0.350	0.364	0.006	0.63

[1]Control, no fat supplement; [2]HVO, supplemented with 3% DM hydrogenated vegetable oil; [3]OO, supplemented with 3% DM olive oil; [4]SEM: standard error of the mean standard error of the mean and values are LSM and pooled SEM.[5]'Others' refers to the less abundant Phylum (relative abundance ≤ 0.5%). Data were analyzed by one-way ANOVA and means were compared by Kruskal-Wallis test. Results were declared significant at P<0.05.

Table S4. PERMANOVA (Weighted) results.

PERMANOVA (Weighted) analysis of the effect of dietary treatments on rumen bacterial diversity

Treatment	Period	SumsOfSqs	MeanSqs	F.Model	R2	Pr(>F)
Control vs. HVO	21 days	0.063	0.063	2.812	0.319	0.052
Control vs. HVO	42 days	0.023	0.023	0.450	0.069	0.760
Control vs. HVO	63 days	0.006	0.006	0.598	0.091	0.658
Control vs. OO	21 days	0.022	0.022	1.717	0.222	0.229
Control vs. OO	42 days	0.017	0.018	1.247	0.172	0.320
Control vs. OO	63 days	0.015	0.015	1.505	0.200	0.255

SumOfSqs, sum of squares; MeanSqs, mean sum of squares; F, F value by permutation; Statistical significance was declared at P<0.05; P-value based on 999 permutations.

Table S5. PERMANOVA (Weighted) results.

PERMANOVA (UnWeighted) analysis of the effect of dietary treatments on rumen bacterial diversity

Treatment	Period	SumsOfSqs	MeanSqs	F.Model	R2	Pr(>F)
Control vs. HVO	21 days	0.248	0.248	1.341	0.183	0.049
Control vs. HVO	42 days	0.216	0.216	0.937	0.135	0.492
Control vs. HVO	63 days	0.167	0.167	0.867	0.126	0.548
Control vs. OO	21 days	0.264	0.264	1.507	0.200	0.062
Control vs. OO	42 days	0.252	0.252	1.439	0.193	0.062
Control vs. OO	63 days	0.214	0.214	1.188	0.165	0.223

SumOfSqs, sum of squares; MeanSqs, mean sum of squares; F, F value by permutation; Statistical significance was declared at P<0.05; P-value based on 999 permutations

Table S6**.** Predicted Functions in Control Diet.

Predicted functions with significant variations in control diet

Predicted functions	*P*- value		
	21 days	42 days	63 days
ABC transporters	0.81	0.99	< 0.01
DNA repair and recombination proteins	< 0.01	0.99	< 0.01
Pores ion channels	0.74	0.25	< 0.01
Protein kinases	0.24	0.04	0.39
Purine metabolism	0.01	0.97	0.12
Pyrimidine metabolism	0.04	0.83	0.99
Ribosome	0.03	0.77	0.73
Transcription factors	0.96	0.88	< 0.01
Transporters	0.99	0.46	< 0.01
Two-component system	< 0.01	< 0.01	< 0.01

Data were analyzed by multiple group ANOVA and Dunnett's multiple comparisons test. Results were declared significant at P<0.01.

Table S7. Predicted Functions in OO Diet.

Predicted functions with significant variations in olive oil diet

Predicted functions	*P*- value		
	21 days	42 days	63 days
ABC transporters	0.93	< 0.01	0.86
Alanine, aspartate and glutamate metabolism	0.99	0.01	0.21
Amino acid related enzymes	0.92	< 0.01	0.05
Amino sugar and nucleotide sugar metabolism	0.99	< 0.01	0.25
Aminoacyl-tRNA biosynthesis	0.88	0.01	0.29
Arginine and proline metabolism	0.98	0.02	0.28
Bacterial motility proteins	0.97	0.01	0.35
Carbon fixation pathways in prokaryotes	> 0.99	0.04	0.34
Chaperones and folding catalysts	0.99	0.02	0.15
Chromosome	0.79	< 0.01	0.22
DNA repair and recombination proteins	0.34	< 0.01	0.02
General function prediction only	0.77	< 0.01	0.15
Glycine, serine and threonine metabolism	0.97	0.04	0.22
Methane metabolism	0.99	< 0.01	0.26
Other ion-coupled transporters	0.70	0.01	0.72
Peptidases	0.89	< 0.01	0.04
Pores ion channels	0.84	0.24	< 0.01
Protein kinases	0.37	< 0.01	0.03
Purine metabolism	0.61	< 0.01	0.02
Pyrimidine metabolism	0.89	< 0.01	0.01
Pyruvate metabolism	0.98	0.03	0.46
Ribosome	0.84	< 0.01	0.01
Ribosome Biogenesis	0.95	< 0.01	0.06
Starch and sucrose metabolism	0.99	< 0.01	0.29
Transcription factors	0.99	< 0.01	0.92
Transcription machinery	0.97	< 0.01	0.03
Transporters	0.97	< 0.01	0.14
Two-component system	0.015	< 0.01	< 0.01

Data were analyzed by multiple group ANOVA and Dunnett's multiple comparisons test. Results were declared significant at P<0.01.

Table S8**.** Predicted Functions in HVO Diet.

Predicted functions with significant variations in hydrogenated vegetable oil diet

Predicted functions	*P*- value		
	21 days	42 days	63 days
ABC transporters	0.44	< 0.01	0.22
Alanine, aspartate and glutamate metabolism	0.09	0.02	0.06
Amino acid related enzymes	< 0.01	< 0.01	< 0.01
Amino sugar and nucleotide sugar metabolism	0.02	< 0.01	0.01
Aminoacyl-tRNA biosynthesis	0.09	0.01	0.05
Arginine and proline metabolism	0.19	0.03	0.13
Chaperones and folding catalysts	0.05	0.02	0.03
Chromosome	0.040	< 0.01	0.02
DNA repair and recombination proteins	< 0.01	< 0.01	< 0.01
DNA replication proteins	0.22	0.05	0.12
General function prediction only	0.04	< 0.01	0.01
Methane metabolism	0.17	0.022	0.12
Other ion-coupled transporters	0.17	0.02	0.09
Peptidases	< 0.01	< 0.01	< 0.01
Pores ion channels	< 0.01	0.02	< 0.01
Protein kinases	0.03	< 0.01	0.01
Purine metabolism	< 0.01	< 0.01	< 0.01
Pyrimidine metabolism	< 0.01	< 0.01	< 0.01
Ribosome	< 0.01	< 0.01	< 0.01
Ribosome Biogenesis	< 0.01	< 0.01	< 0.01
Starch and sucrose metabolism	0.02	< 0.01	0.01
Transcription factors	0.18	< 0.01	0.19
Transcription machinery	0.02	0.01	0.01
Transporters	< 0.01	< 0.01	< 0.01
Two-component system	< 0.01	< 0.01	< 0.01

Data were analyzed by multiple group ANOVA and Dunnett's multiple comparisons test. Results were declared significant at $P<0.01$.

Table S9. KEGG classification of functional gene categories

Level 1	Level 2	Level 3	Database
Environmental Information Processing	Membrane transport	ABC transporters	PATHWAY
	Signal transduction	Two-component system	PATHWAY
Genetic Information Processing	Translation	Aminoacyl-tRNA biosynthesis	PATHWAY
		Ribosome	PATHWAY
	Transcription	Transcription factors	PATHWAY
Metabolism	Amino Acid Metabolism	Alanine, aspartate and glutamate metabolism	PATHWAY
		Glycine, serine and threonine metabolism	PATHWAY
		Arginine and proline metabolism	PATHWAY
	Carbohydrate Metabolism	Amino sugar and nucleotide sugar metabolism	PATHWAY
		Pyruvate metabolism	PATHWAY
		Starch and sucrose metabolism	PATHWAY
	Energy Metabolism	Carbon fixation pathways in prokaryotes	PATHWAY
		Methane metabolism	PATHWAY
	Nucleotide Metabolism	Purine metabolism	PATHWAY
		Pyrimidine metabolism	PATHWAY
Genes and proteins	Genetic Information Processing	DNA repair and recombination proteins	BRITE
		Chaperones and folding catalysts	BRITE
		Chromosome	BRITE
		Ribosome Biogenesis	BRITE
		Transcription machinery	BRITE
		DNA replication proteins	BRITE
	Signaling and Cellular Processes	Transporters	BRITE
		Bacterial motility proteins	BRITE
	Metabolism	Protein kinases	BRITE
		Amino acid related enzymes	BRITE
		Peptidases	BRITE
Unclassified	Cellular Processes and Signaling	Pores ion channels	BRITE
		Other ion-coupled transporters	BRITE
	Poorly Characterized	General function prediction only	BRITE
